# Appraising the role of visual threat in speeded detection and classification tasks

**DOI:** 10.3389/fpsyg.2015.00755

**Published:** 2015-06-16

**Authors:** Yue Yue, Philip T. Quinlan

**Affiliations:** ^1^Department of Psychology, Liverpool Hope UniversityLiverpool, UK; ^2^Department of Psychology, University of YorkYork, UK

**Keywords:** visual threat detection, object classification, speeded visual search, fear response hypothesis, search efficiency

## Abstract

This research examines the speeded detection and, separately, classification of photographic images of animals. In the initial experiments each display contained various images of animals and, in the detection task, participants responded whether a display contained only images of birds or also included an oddball target image of a cat or dog. In the classification search task, a target was always present and participants classified this as an image of a cat or a dog. Half of the target images depicted the animal in a non-threatening state and the remaining half images depicted the animal in a threatening state. A complex pattern of effects emerged showing some evidence of more efficient detection of a threatening than non-threatening target. No corresponding pattern emerged in the data for the classification task. Next the tasks were repeated when the stimuli were more carefully matched in terms of general pose and salience of facial features. Now the effects in the detection task were reduced but more consistent than before. Threatening targets were more readily detected than non-threatening targets. In addition, non-threatening targets were more readily classified than threatening targets. The nature of these effects appears to reflect decisional/response mechanisms and not search processes. The performance benefit for the non-threatening images was replicated in a final classification task in which, on each trial, only a single peripheral image was presented. The results demonstrate that a number of different affective and perceptual factors can influence performance in speeded search tasks and these may well be confounded with the variation in threat content of the experimental stimuli. The evidence for the automatic detection of visual threat remains illusive.

## Introduction

The present experiments were primarily motivated to test the *fear response hypothesis* as put forward by Öhman (1999) and Öhman and Mineka (2001). The hypothesis states that humans have evolved a *fear system* (Öhman and Mineka, 2001), which is rapidly and automatically elicited by the presence of a threat in the immediate environment. The fear system should be invoked whenever a threat confronts an observer. Its prime purpose is to produce an automatic early warning signal that alerts the observer to the threat. A further claim is that the fear system can become activated even though the associated stimulus has not been fully analyzed. The system can be alerted to a threat even though the nature of the threat object has not been determined. These claims are examined in detail in the experiments reported here.

In addressing the fear response hypothesis, primary interest has been with whether participants show an advantage in detecting the presence of an image of a threatening item in a display containing images of non-threatening items gauged relative to detecting a non-threatening item in the presence of threatening items. Such an advantage – known as the *threat advantage* (see Quinlan and Dyson, 2008, Chapt. 16) – has been examined in various kinds of speeded visual search tasks. For instance, Öhman et al. (2001) used an oddball version of the search task in which each search item was a full color photographic image of a plant or an animal, and participants, on each trial, had to judge whether all the search items were taken from the same biological category or whether there was a distinctive singleton (an oddball) present. Two categories of, so-called, fear-relevant items (henceforth threatening items) were chosen, namely, spiders and snakes, and two categories of fear-irrelevant items (henceforth, non-threatening items) were also chosen, namely, flowers and mushrooms.

Across trials, the display size could be either four or nine items and the threat advantage was examined as a function of display size. It was found that, statistically, reaction time (RTs) did not increase directly with increases in display size when the target was a threatening item: the time to detect the threatening target was, statistically, the same regardless of how many items were to be searched. However, RTs did increase directly as a function of display size when the target was a non-threatening item. Critically, the numerical increase in RTs with display size for the threatening targets showed an additional 3 ms time cost for each search item in the display: a slope value which has been traditionally associated with parallel search (e.g., Treisman and Souther, 1985, p. 471). This very shallow slope suggests that threat detection was automatic.

Following on from the work of Öhman et al. (2001), there has been some further work on the influence of visual threat in speeded search tasks (e.g., Lipp et al., 2004; Brosch and Sharma, 2005; Blanchette, 2006). The empirical consequences have been evaluated, and the relevant evidence has turned out to be somewhat controversial (see Quinlan, 2013, for a review). For instance, there are some serious concerns about possible stimulus factors that are confounded with the variation in threat content of the search stimuli. For example, Tipples et al. (2002) replicated the experiment conducted by Öhman et al. (2001, Experiment 2), and found the threat advantage in searching for threatening animals amongst plants. Furthermore, Tipples et al. (2002) found the same detection advantage in searching for the non-threatening animals amongst plants. Collectively, the data were taken to suggest that the original threat advantage was confounded with the animal/plant distinction and that the actual target detection advantage was due to better detection of animals than of plants, regardless of the emotional content of the images.

LoBue and DeLoache (2008, 2011) considered the influence of animal distinctiveness, and took some steps to avoid confounding variables. Search for a distinctive snake amongst frogs was compared with search for a distinctive frog amongst snakes. The results of this experiment revealed a strong snake advantage, such that the detection of a snake target was easier than the detection of a frog target. Hence the detection of a distinctive animal was not in itself sufficient to explain why images of snakes were easier for participants to respond to than images of frogs. LoBue and DeLoache (2008, 2011) acknowledged that the very distinctive, elongated, limbless shape of the snake contributed to its rapid detection.

It is also notable that, in the studies by LoBue and DeLoache (2008), the non-targets in the search displays differed according to which target was being searched for. For example, participants searched through displays containing images of frogs in a bid to find an image of a snake and they searched through displays containing images of snakes in a bid to find an image of a frog. Given this, it is impossible to disentangle effects due to target detection from those concerned with rejecting non-targets. The difficulty of locating the target frog image may reflect the difficulty of searching through the non-target snake images. Given these concerns it is important to consider possible confounds in designing new speeded search tasks that implicate threat processing. For instance, the basic target/non-target categorical differences should be controlled for. In addition, the same non-targets should be used with both threatening and non-threatening targets.

A notable feature of the oddball version of the visual search task is that participants never have to identify the nature of the target threat: The task is to ascertain merely whether a target singleton is present or not. A concern with the original experiments (e.g., Öhman et al., 2001, Experiment 1) is that the target was distinctive in being the only animal image in the display and also in being the only threatening image in the display. We might ask about the degree to which the threat advantage is due to speed of detection, speed of identification or both. Such a question is particularly pertinent, because, according to the fear response hypothesis, the fear response can be invoked even though the nature of the actual threat has yet to be identified.

In exploring such issues we compared target detection and target classification in two speeded visual search tasks based on the oddball task described by Öhman et al. (2001). In the detection task, participants merely had to judge whether an image of a distinctive target animal was present on each trial. Each of the search items was a photograph that contained the image of a single animal. On a random half of the trials no target image was present. Participants were simply instructed to press one key if the search display contained images only of birds and to press a different key if a distinctive image of an animal other than a bird (in this case, a dog or cat) was present. In contrast, in the classification task, on every trial a target image was present and participants had to classify what kind of animal the target was. They simply had to press one key if the target image was of a dog and a different key if the target image was of a cat.

In both cases the non-target images were of well-known wild birds (not birds of prey). In this way all target images were present against the same kind of neutral non-target images. Half the target images were of dogs and half were of cats and for both of these categories, half of the images depicted threatening animals and half depicted non-threatening animals. Therefore, there was no category confound between the threatening and non-threatening instances (cf. Quinlan, 2013). Moreover, in neither of these tasks was the emotional valence of the target image a cue to response, hence any effects of emotional valence – in this case, ‘threat’ – cannot be due to some form of response priming.

The experiments were motivated by two primary aims namely, (1) to establish firm evidence for a threat advantage when more careful experimental controls had been introduced, and (2) to provide direct support for a basic claim of the fear response hypothesis that threat items can be readily detected prior to being fully identified.

## Experiment 1

Two tasks were designed, namely, (i) a present/absent speeded search task (henceforth, the *detection task*), and (ii) a cat/dog classification task (henceforth the *classification task*). The same sorts of visual displays were used in both tasks. On every trial, the participant was presented with a visual display containing three, six, or nine colored photographs. In both tasks, the same photographs were used and the non-target photographs were of single wild birds (not birds of prey). The target set of photographs were divided into four sets namely, non-threatening dogs (i.e., dogs from domesticated breeds), threatening dogs (e.g., wolves, hyenas, attack dogs depicted in a threatening disposition), non-threatening cats (i.e., cats from domesticated breeds), and, threatening cats (i.e., wild cats – lions, tigers, panthers – depicted in a threatening disposition). All threatening animals were shown snarling.

According to the fear response hypothesis, the basic prediction is that detection responses should be faster on threatening target trials (on trials where the target is an image of a threatening animal) than on non-threatening target trials (on trials where the target is an image of a non-threatening animal). This is despite the fact that the valence of the target images is incidental to the response. Of additional interest is the degree to which effects are mirrored in the data for the classification task.

In the classification task, participants were instructed to classify the target as either a dog or cat and, again, the emotional valence of the images (threatening vs. non-threatening) had no bearing on this decision. Nonetheless, on the grounds that threatening images are easier to detect than the non-threatening images, then it would seem plausible that any benefits that might accrue during search are preserved at the level of target classification. At the very least, therefore, it could be argued that the same effects of threat found in the detection data ought to be present in the data for the classification task. Any differential effects of threat across the two tasks would then suggest that the influence of threat varies according to the task constraints.

### Method

#### Stimuli

Each photograph (i.e., each search item) depicted a single animal in its natural habitat rendered in full color. Each photograph was 5.5° (wide) × 3.5° (high) visual angle. Photographs were arrayed around a virtual circle whose radius was 8.5°. When nine photographs were presented the photographs were spaced at the apexes of a nine-sided polygon. These nine screen positions acted for the photographic place holders for all of the displays. For the smaller display sizes, the item positions were chosen at random from these nine place holders prior to each trial. The individual pictures were sourced from various Internet searches and some items were taken from the International Affective Picture System (IAPS; see Lang et al., 2005). Each of the four target sets of photographs (threatening/non-threatening cats and dogs) comprised 48 different items. The non-target items for a given display were selected at random (without replacement) prior to each trial from a basic set of 421 items.

To assess threat content of the images, an independent group of 24 undergraduate participants rated all of the target images for valence and separately for arousal. A 7-point Likert scale accompanied each image. For the threat judgments 1 represented non-threatening and 7 represented very threatening. For the arousal judgments 1 represented not arousing and 7 represented very arousing. The average threat ratings were, mean = 5.57, SD = 0.31; mean = 5.57, SD = 0.39; mean = 2.04, SD = 0.41; mean = 2.05, SD = 0.48; for the threatening cats, threatening dogs, non-threatening cats, non-threatening dogs, respectively. The ratings were entered into a two-way repeated measures ANOVA in which valence (threatening vs. non-threatening) and animal (cat vs. dog) were entered as fixed factors and participants acted as a random factor. Only the main effect of valence reached statistical significance, *F*(1,47) = 2939.47, MSE = 0.203, *p* < 0.001. Consequently, the dog and cat images were equivalent in terms of rated threat and the threatening images were rated as being reliably more threatening than the non-threatening images.

The average arousal ratings were, mean = 5.29, SD = 0.25; mean = 5.24, SD = 0.34; mean = 2.69, SD = 0.33; mean = 2.69, SD = 0.37; for the threatening cats, threatening dogs, non-threatening cats, non-threatening dogs, respectively. These ratings were analyzed in the same way as the threat ratings and again only the main effect of valence reached statistical significance, *F*(1,47) = 2641.85, MSE = 0.12 *p* < 0.001. Consequently, the threatening images were rated as being more arousing than the non-threatening images and there were no further effects due to animal category.

#### Design

In the detection task, the experimental trials were divided into four blocks. Within each block there were 96 trials in total, 48 trials contained a target (henceforth were Present trials) and 48 contained no target (henceforth were Absent trials). Within the Present trials there were 16 trials for each display size and, for each display size, there were four items chosen from each of the four target sets. Corresponding Absent trials were configured. Each participant saw each target only once in the detection task and the allocation of targets to the particular display sizes was randomized across participants. There were 384 experimental trials in total and the order of the trials within the blocks was randomized for each participant. Prior to the experimental trials there was a single block of 24 practice trials and the targets in these practice trials were different from the targets in the experimental trials.

In the classification task, each display contained a target and the structure of the blocks was the same as in the detection task. There were, however, no Absent trials. The participants’ task was to classify the target as depicting either a dog or a cat. There were 48 trials per block and half contained a photograph of a dog and half contained a photograph of a cat. Half of the targets were threatening images and half were non-threatening images. Within each block, there were 16 trials for each display size and for each display size there were four items chosen from each of the four target sets. The balancing and allocation of the items to the displays was as in the detection task. In this case, there were 192 experimental trials and a single block of 12 practice trials was administered prior to the experimental trials. No target item was presented more than once in the classification task and the same target items were used as in the detection task.

Half of the participants carried out the detection task prior to the classification task and for the remaining participants this order was reversed. In the detection task, half of the participants responded Present with a left key press and half responded Present with a right key press. In the classification task half of the participants responded to DOG with a left key press and half responded to DOG with a right key press.

#### Apparatus

The E-prime program (Schneider et al., 2002), running on a Windows 2000 PC, was used for controlling the experiments. In addition, an E-prime response box was used to collect the responses. Keys 1 and 2 were used throughout. Stimulus delivery was via a 15″ SONY monitor (model CPD-100ES). Auditory trial feedback when an error was committed (i.e., a standard beep) was delivered via headphones.

#### Participants

Twenty-four naïve participants (mean age = 22, 18 female) were recruited from students of York University. There were two left-handed individuals. They received either a course credit or £4. All of the participants reported normal or corrected-to-normal vision.

#### Procedure

Each participant was tested individually in a quiet, window-less, testing cubicle. Participants were seated at a table in front of a chin rest situated 57 cm from a computer screen which was located on a raised plinth. The center of the screen was at eye-level. On the table in front of the screen was placed the E-prime button box. The screen and response box were linked to a PC computer situated outside the cubicle.

Initially participants were provided with task instructions and the response allocation was described. They were also told that response timing began once the search display was presented and that they had to respond to as quickly and as accurately as possible.

Participants initiated a block of trials with any response key press. Every trial began with the presentation of a central fixation mark (i.e., a “+”) for 600 ms. At the offset of the fixation plus, the search display was immediately presented. The display remained on until the computer detected a key press response. Whenever an error was committed the computer issued a beep. The inter-trial interval was set at 1 s. In total the detection tasks lasted approximately, 25 min and the classification task lasted 15 min. Both tasks were completed in a single testing session.

### Results

#### Detection Task

See **Table 1** for a summary of the RTs and error rates per condition.

**Table 1:** Mean reaction times (RTs), SE, and mean percentage error rates (%E) for the various conditions of interest in the detection task in Experiment 1.

	Display set size								
	3			6			9			
Trial type	Mean	SE	%E	Mean	SE	%E	Mean	SE	%E
Threatening cat	851	21	3.6	968	28	2.9	1056	25	4.2
Threatening dog	869	30	4.4	975	23	4.4	1141	26	5.7
Non-threatening cat	1020	31	1.2	1129	14	9.6	1297	39	14.8
Non-threatening dog	905	24	8.1	1104	27	7.3	1279	37	12.2
Absent	1000	13	1.3	1512	33	1.3	1852	56	1.8

The raw data, for the various conditions of interest, were transformed into mean RTs and error rates. These data were then used to compute inverse efficiency (IE) scores for each participant for each condition of interest. An IE score is defined as the average RT divided by the proportion correct for a particular condition. IE scores were first discussed as providing useful indices of information processing performance by Townsend and Ashby (1983). IE scores have been used in a number of different contexts (Spence et al., 2001; Goffaux et al., 2005; Shore et al., 2006) and, in particular, in studies of threat detection in speeded visual search tasks (see Godwin et al., 2010). Here they allow us to convey the key findings succinctly. The RT data and errors were also analyzed in a comparable fashion and the key effects, as reported here, emerged in the RT analyses. Error rates were generally low across all the experiments and were overwhelmingly less than 10%/condition. Mean RTs and summary error data are reported in the accompanying tables. There is no evidence of any systematic speed/accuracy trade-offs in any of the experiments. Increasing task difficulty is reflected in slower RTs and less accurate responding: IE scores directly reflect this.

**FIGURE 1:**
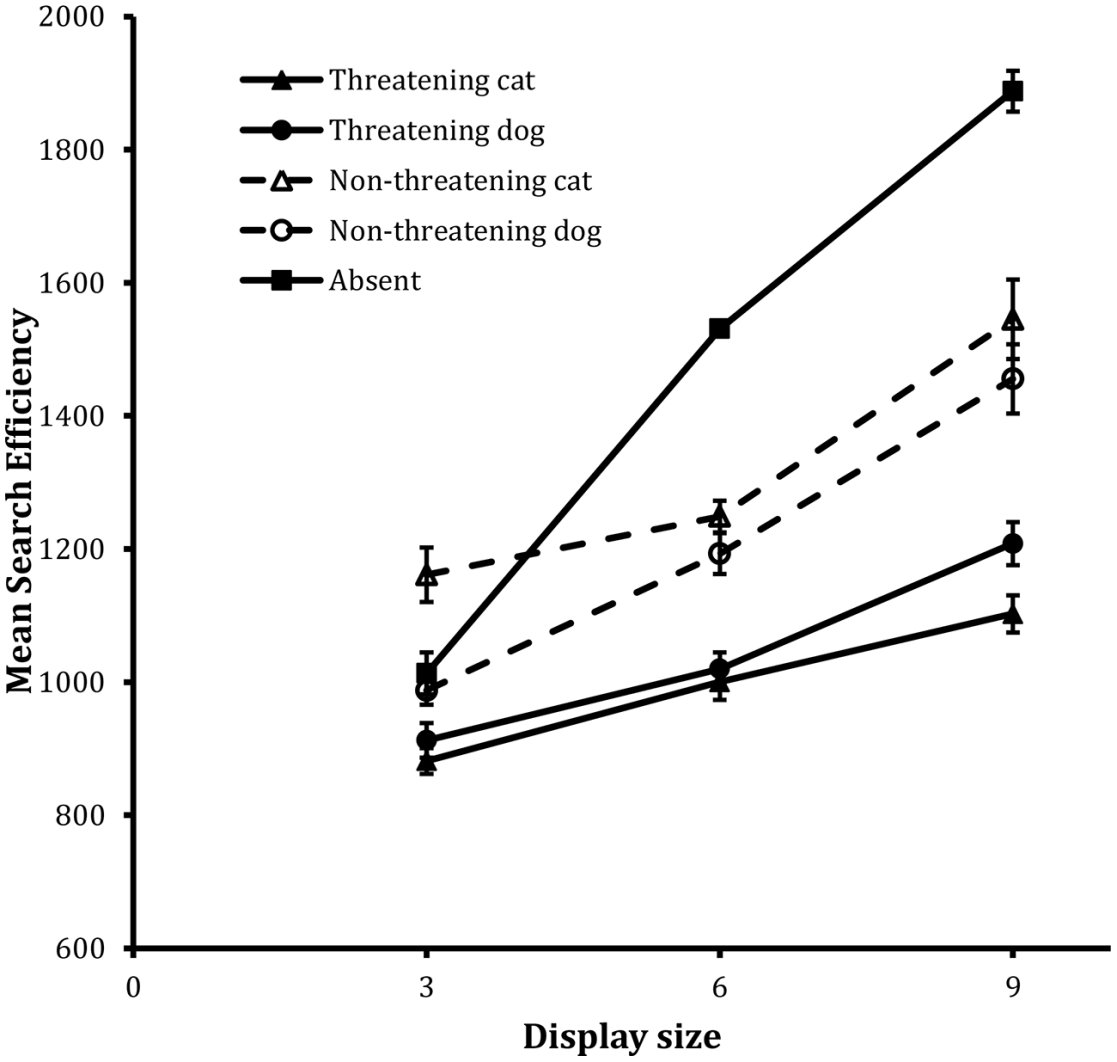
**Mean search efficiency scores as a function of both condition and display set size in the detection task in Experiment 1**. Error bars reflect within-participant SE after variation between participants was removed (after Bakeman and McArthur, 1996).

**Figure 1** shows the search functions from the trials for the four target types together with that for the Absent trials computed from the associated IE scores. Data for Present and Absent trials are dealt with separately, in turn.

##### Present trials

The IE scores were entered into a 2 × 2 × 3 repeated-measures ANOVA in which emotional valence (henceforth, valence: threatening vs. non-threatening), animal (cat vs. dog) and display size (3, 6, 9 items) were entered as fixed factors and participant was entered as a random factor. The analysis revealed statistically significant main effects of valence, *F*(1,23) = 116.03, MSE = 37060, *p* < 0.001, and display size, *F*(2,46) = 68.41, MSE = 41849, *p* < 0.001.

Two-way interactions between valence and animal, *F*(1,23) = 21.12, MSE = 21363, *p* < 0.001, and valence and display size, *F*(2,46) = 6.26, MSE = 30135, *p* < 0.01, were also statistically reliable. To examine the valence × animal interaction further, a Tukey’s HSD test was carried out on the corresponding marginal means. This revealed reliable effects of valence on both cat and dog target trials (both *p*s < 0.05) and inspection of the data revealed that the valence effect was larger on the cat target trials (323 units) than the dog target trials (165 units, see also **Figure 1**).

The valence × display size interaction is best explained in the following terms. Both functions were well described by a linear component (*r^2^* = 0.99 and 0.97, respectively), but the search function for the non-threatening target trials possessed a steeper slope (i.e., 71 units/item) than the search function for the threatening target trials (i.e., 43 units/item).

In summary, the data on present trials revealed that, overall, search efficiency was greater on threatening target trials than on non-threatening target trials. The threat advantage was also revealed in the slopes of the corresponding functions. That is, the decrement in search efficiency with increases in display size was greater on non-threatening target trials than on threatening target trials. In addition, the effects of threat were more pronounced on cat target trials than on dog target trials, as a function of display size.

##### Absent trials

The analysis of the IE scores revealed that, although the Absent search function was well described by a linear trend, *F*(1,23) = 201.91, MSE = 45468, *p* < 0.001, for the linear component, the departure from linearity was also statistically reliable, *F*(1,23) = 42.02, MSE = 2458, *p* < 0.001, for the quadratic component. This trend is clear from visual inspection of ?? and is similar to effects reported widely in the speeded visual search literature (see e.g., Quinlan and Humphreys, 1987; Chun and Wolfe, 1996).

#### Classification Task

See **Table 2** for a summary of the RTs and error rates per condition.

**Table 2:** Mean RTs, SE, and mean percentage error rates (%E) for the various conditions of interest in the classification task in Experiment 1.

	Display set size								
	3			6			9			
Trial type	Mean	SE	%E	Mean	SE	%E	Mean	SE	%E
Threatening cat	1043	23	9.6	1202	24	9.4	1332	32	9.1
Threatening dog	1148	22	6.8	1339	26	7.8	1477	41	7.3
Non-threatening cat	1059	26	2.1	1346	28	2.9	1608	42	2.9
Non-threatening dog	1127	26	3.4	1297	32	2.9	1500	34	5.7

**FIGURE 2:**
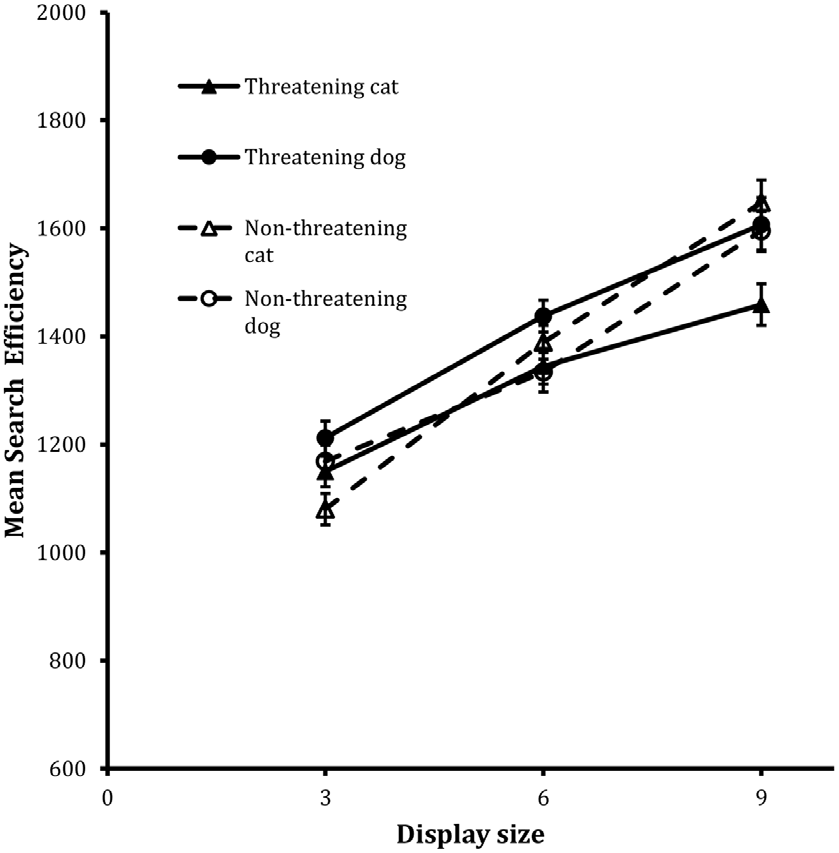
**Mean search efficiency scores as a function of both condition and display set size in the classification task in Experiment 1**. Error bars reflect within-participant SE after variation between participants was removed (after Bakeman and McArthur, 1996).

**Figure 2** shows the search functions from the trials for the four target types. The corresponding IE scores were entered into the same kind of 2 × 2 × 3 repeated-measures ANOVA as used with the Present scores in the detection task. The analysis revealed statistically significant main effects of animal, *F*(1,23) = 4.68, MSE = 34604, *p* < 0.05, and, display size, *F*(2,46) = 112.98, MSE = 38300, *p* < 0.001. The main effect of animal revealed that performance on the cat target trials was more efficient than it was on the dog target trials.

In addition, the two-way interactions between valence and animal, *F*(1,23) = 7.19, MSE = 28885, *p* < 0.05, and between valence and display size, *F*(2,46) = 4.12, MSE = 34737, *p* < 0.05, were also statistically reliable. Finally, the three way interaction between valence, animal and display size exhibited a trend toward statistical significance, *F*(2,46) = 2.71, MSE = 31181, *p* = 0.07.

In order to examine the higher-order effects in more detail, separate analyses were carried out on the data from the cat and dog target trials respectively. In both cases, 2 × 3 ANOVAs were carried out in which the valence, display size and participant factors were the same as before. For the cat target trials, the analysis showed that both the main effect of valence, *F*(1,23) = 4.64, MSE = 230037, *p* < 0.05, and display size, *F*(2,46) = 76.93, MSE = 30164, *p* < 0.001, were statistically reliable as was the valence × display size interaction, *F*(2,46) = 6.12, MSE = 32953, *p* < 0.01. Inspection of the slopes of the corresponding search functions revealed that the effect of display size was more marked in the data for the non-threatening targets (i.e., 95 units/item) than the threatening targets (i.e., 52 units/item).

The analysis of the dog target trials revealed a different pattern of performance. In this case, the only test to reach statistical reliability was associated with the main effect of display size, *F*(2,46) = 62.11, MSE = 32620, *p* < 0.001.

Overall therefore the data show reliable effects of display size in all cases. However, the effects of valence are attributable to performance on the cat target trials. The size of the threat advantage scaled with increases in display size on the cat target trials. There were no corresponding effects of valence in the data for the dog target trials.

### Discussion

The results of the detection experiment are generally in line with the fear response hypothesis. There was a clear and overall threat advantage – threatening targets were processed more efficiently than non-threatening targets. In addition to this main effect of valence, the threat advantage was also expressed in the valence by display size interaction. Threatening targets were generally detected more efficiently than non-threatening targets and the detection of non-threatening targets was particularly affected by increases in display size. The overall effect of valence was greater on the cat target trials than the dog target trials. This reveals that participants performed relatively poorly in detecting the non-threatening cat images.

There is, however, no indication that performance on target present trials is in line with the notion of target pop-out. Inspection of the search rates computed from the mean RTs for the corresponding cases reveals a slope value of 34.1 ms/item for the threatening cat target trials and a value of 45.3 ms/item for the threatening dog target trials. Both of these values are substantially greater than 10 ms/item that has been used to define “efficient” searches (Wolfe, 1998) or, indeed, the 5 ms/item that has been used to define target pop-out (Treisman and Souther, 1985).

In the detection task, the pick-up of any kind of distinctive cue associated with the target was sufficient to make a response. We have taken the view that the only consistent difference across the threatening and non-threatening targets was the presence/absence of the snarling facial configuration. If we accept the fear response hypothesis, then the assumption is that participants are particularly sensitive to the presence of this sort of threat cue. Consequently, it is the pick-up of the snarling facial cues that alerts participants to the likelihood of the presence of a target and because of this the threat advantage obtains.

This, however, is not the only possible account of performance because the data show that other stimulus factors also played a role. The nature of the valence effects in the detection task varied across the cat and dog target trials – the effects were larger in the data for the cat target trials than they were in the dog target trials. For whatever reason the non-threatening cat targets were particularly difficult to detect. There are no obvious reasons for these stimulus-specific differences and this appears to underscore the fact that in such speeded search tasks, participants may respond on the basis of detecting any one of a variety of discriminating visual cues.

Nonetheless, the data do indicate the presence of interesting dissociations between the apparent effects of threat in the detection task and the influence of the valence in the classification data. The effects of threat are generally clear-cut in the detection task but they are mixed in the data for the classification task. Whereas there is no overall difference in the processing of the threatening and non-threatening dog images, the effects of threat with the cat images were inconsistent. The data show that at the lowest display size the threatening images were processed no more efficiently than the non-threatening images (if anything the reverse occurred), but at the largest display size the threat effect emerged. There is no obvious reason for this particular pattern of findings but nonetheless, they do indicate that threat effects found in the detection task are only imperfectly reflected in the classification performance.

In order to examine the generalizability of the effects, now a much more careful approach was taken to image selection so as to try and control for any incidental differences between the threatening and non-threatening images. In selecting the threatening target images for the first experiment, the defining criterion was that the animal be pictured snarling. Given this, the overwhelming majority of the threatening target images were of headshots of the animals. In contrast, and by necessity, because this criterion had not been applied to the selection of the non-threatening target images, there was much more variety in the poses of the animals captured in the non-threatening target images. There were 10 and 12 headshots of the non-threatening cats and dogs, respectively, and 34 and 38 headshots of the threatening cats and dogs, respectively. Maybe, therefore, the presence of the target was easier to detect when a headshot was presented than when facial features in the image were less salient? This is not an entirely speculative proposition given that, at least, when human faces are used, participants are able to extract emotional expression information rapidly from images of faces presented in peripheral vision (see Goren and Wilson, 2006; Haberman and Whitney, 2007; Calvo and Nummenmaa, 2008; Calvo et al., 2010).

In a bid to address this possibility, a more careful selection of the non-threatening target images was undertaken. Controls were undertaken to match up the threatening and non-threatening targets so that in both cases the facial features and general pose of the animals in the images were similar. The original threatening images used in the prior experiments were retained, but matching to these images was undertaken in selecting the new non-threatening target images.

## Experiment 2

In all respects – except stimuli and participants – Experiment 2 was the same as Experiment 1. In this case the threatening images were as before and new non-threatening images were chosen so as to control for general pose across the image sets.

### Participants

Twenty-four naïve participants (mean age = 21, 17 female) were recruited from students of York University. There were five left-handed individuals. They received either a course credit or £4. All of the participants reported normal or corrected-to-normal vision.

### Results

#### Detection Task

**FIGURE 3:**
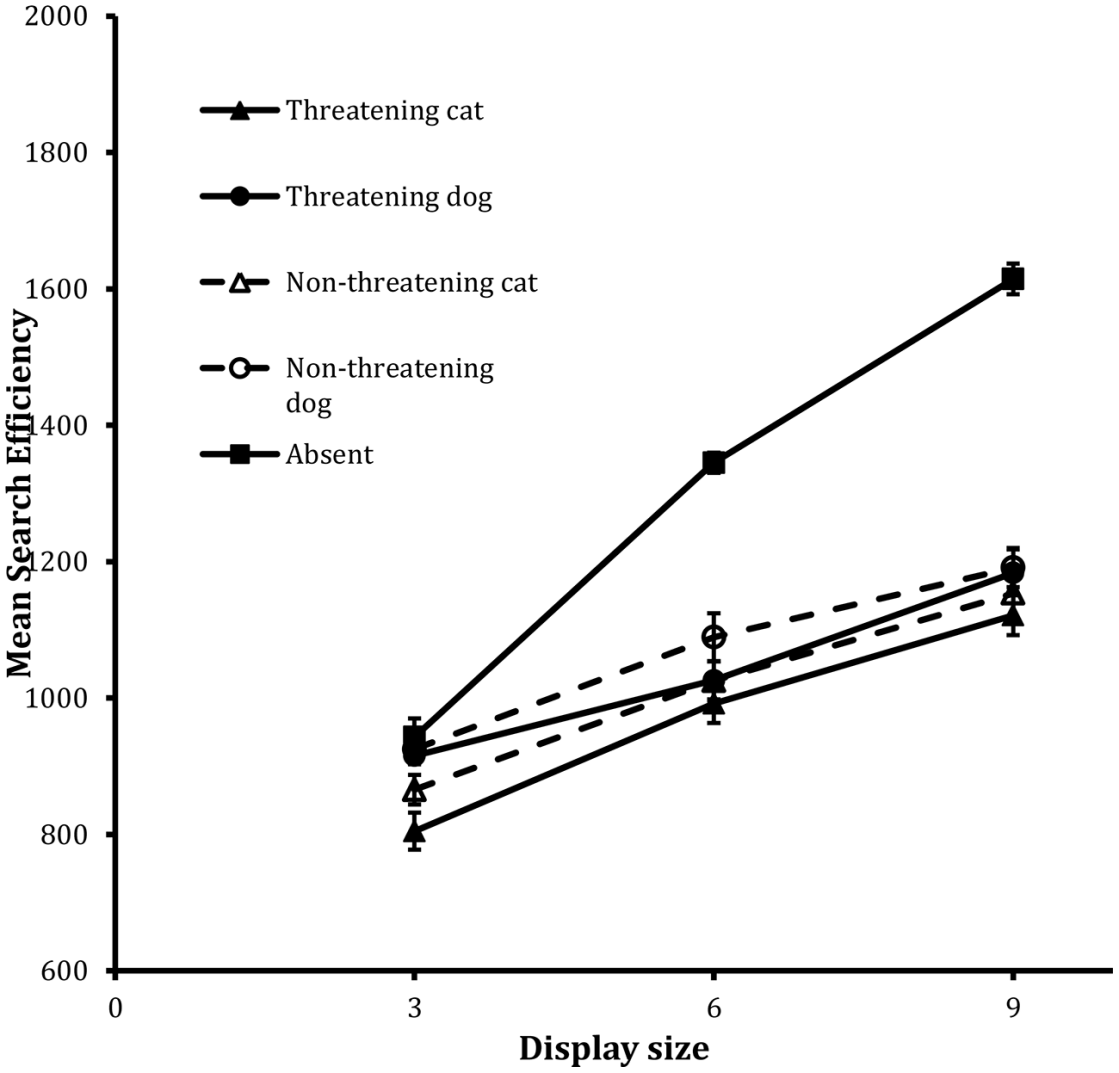
**Mean search efficiency scores as a function of both condition and display set size in the detection task in Experiment 2**. Error bars reflect within-participant SE after variation between participants was removed (after Bakeman and McArthur, 1996).

The same methods of data analysis used before were used here. See **Table 3** for a summary of the RTs and error rates per condition. **Figure 3** shows the search functions from the trials for the four target types together with the function for the Absent trials. Initially interest is with the data for the Present trials.

**Table 3:** Mean RTs, SE, and mean percentage error rates (%E) for the various conditions of interest in the detection task in Experiment 2.

	Display set size								
	3			6			9			
Trial type	Mean	SE	%E	Mean	SE	%E	Mean	SE	%E
Threatening cat	773	30	3.6	937	24	5.7	1045	20	6.3
Threatening dog	837	17	8.3	950	21	7.0	1083	26	8.1
Non-threatening cat	804	17	6.0	968	22	5.5	1037	30	9.4
Non-threatening dog	875	15	5.2	1009	23	6.5	1071	23	9.9
Absent	922	12	2.1	1330	34	1.1	1583	41	1.9

##### Present trials

The analysis revealed statistically significant main effects of valence, *F*(1,23) = 8.12, MSE = 10544, *p* < 0.01, animal, *F*(1,23) = 13.33, MSE = 20302, *p* = 0.001, and display size, *F*(2,46) = 92.48, MSE = 21100, *p* < 0.001. No other tests reached statistical significance.

The main effect of valence revealed an overall threat advantage in the data: performance on threatening target trials was more efficient than it was on non-threatening target trials. Performance was also more efficient on the cat target trials than the dog target trials and again decreases in efficiency scaled with increases in display size.

##### Absent trials

The analysis of the IE scores revealed that, although the Absent search function was well fit by a linear trend, *F*(1,23) = 201.16, MSE = 26914, *p* < 0.001, for the linear component, as in Experiment 1, the departure from linearity was also statistically reliable, *F*(1,23) = 8.96, MSE = 7859, *p* < 0.01, for the quadratic component.

#### Classification Task

See **Table 4** for a summary of the RTs and error rates per condition.

**Table 4:** Mean RTs, SE, and mean percentage error rates (%E) for the various conditions of interest in the classification task in Experiment 2.

	Display set size								
	3			6			9			
Trial type	Mean	SE	%E	Mean	SE	%E	Mean	SE	%E
Threatening cat	1030	14	8.6	1206	26	8.6	1315	31	7.8
Threatening dog	1127	22	8.1	1325	29	5.2	1407	31	8.3
Non-threatening cat	945	21	2.6	1112	25	1.8	1279	27	3.9
Non-threatening dog	1093	26	3.6	1242	22	2.6	1382	27	2.9

**FIGURE 4:**
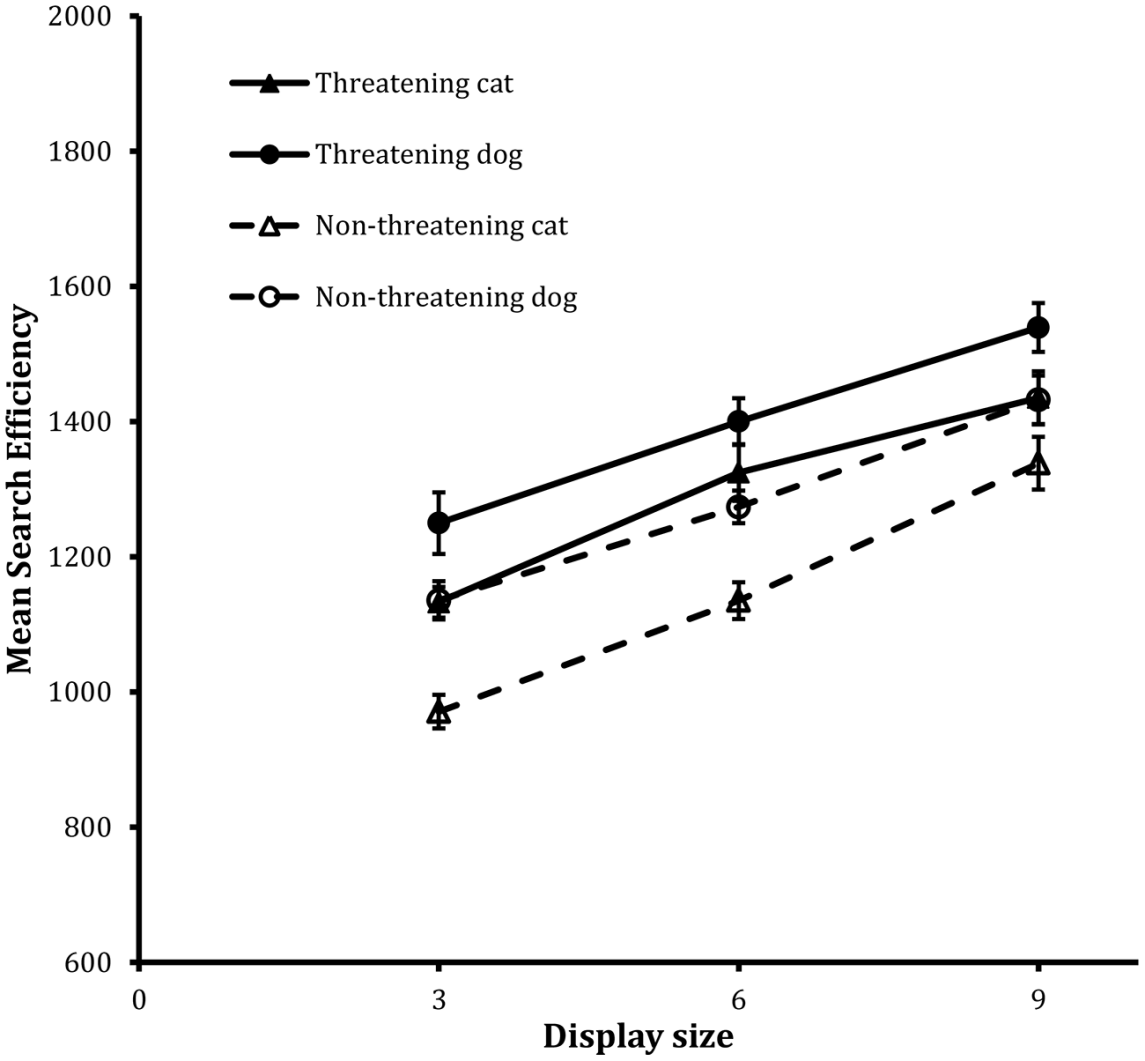
**Mean search efficiency scores as a function of both condition and display set size in the classification task in Experiment 2**. Error bars reflect within-participant SE after variation between participants was removed (after Bakeman and McArthur, 1996).

**Figure 4** shows the search functions from the trials for the four target types. The analysis revealed statistically significant main effects of valence, *F*(1,23) = 38.20, MSE = 33193, *p* < 0.001, animal, *F*(1,23) = 20.75, MSE = 46363, *p* < 0.001, and display size, *F*(2,46) = 85.29, MSE = 43472, *p* < 0.001.

In this case, the most striking thing was that the main effect of valence was quite unlike those reported previously, because now the effect was manifest as a threat disadvantage: performance was more efficient on non-threatening target trials than it was on threatening target trials. Subsidiary to this general pattern, the main effect of animal revealed that performance was better on cat target trials than on dog target trials. Finally the main effect of display size again showed that decreases in efficiency scaled with increases in display size.

### Discussion

The key finding in the detection task is that responses to threatening images were more efficient than were responses to the non-threatening images. Comparing across ?? it clearly is the case that the threat advantage in the detection tasks changed dramatically across the two sets of target images. When more careful controls were undertaken – as in Experiment 2 – so that the facial features and general pose of the animals were matched across the threatening and the non-threatening sets, then the size of the threat advantage decreased dramatically. In following the advice of Bakeman (2005), the effect size of each of the main effects of valence in the two detection tasks was computed via the $\eta_{G}^{2}$ statistic. The effect sizes were 0.27, and 0.06, in Experiments 1 and 2, respectively. What this shows is that when possible visual confounds across the threatening and non-threatening targets were more tightly controlled, then the effects of “threat” were, accordingly, attenuated.

Of particular interest is that the current effects of threat are expressed in terms of intercept differences rather than slope difference. As an additional check, the RT scores were analyzed in the same way as the efficiency scores and exactly the same pattern of effects arose. That is, when response speed was considered, the corresponding search functions for the different target trials revealed intercept and not slope differences. This suggests that the effects reflect non-search processes. A suggestion is that the time to find the target did not vary according to threat content but that the time to respond to the content did. Participants were simply quicker to respond to the threatening than the non-threatening images. This in turn may be linked to the fact that the threat images were more arousing than the non-threatening images (cf. Lundqvist et al., 2014).

Aside from this, the most striking patterns of performance relate to the data from the classification task. In this case, participants were less efficient in responding to the threatening targets than the non-threatening targets. The data revealed robust reverse effects of threat. Indeed such a reverse pattern contrasts with the threat advantage found in the corresponding detection task. Whereas there was a threat advantage when participants responded to the presence of a distinctive target, there was a reverse threat effect when participants were asked to search for and classify the distinctive target.

This reverse threat effect was unexpected but was consistent across all display sizes and for both the cat and dog images. This effect was also expressed as an intercept and not a slope effect. Indeed when the corresponding RT data were analyzed in the same manner as the efficiency scores then the same pattern of statistical significance arose. What this again suggests is that the time to search for the target did not differ across the different target types but the time to respond to them did. In order to test this simple idea a final experiment was carried out in which the classification task was repeated but in a non-search version of the paradigm. In this case only a single image was presented on each trial. The image occupied one of the previous peripheral image locations used in Experiments 1 and 2 and this was chosen at random prior to the start of the trial. If the reverse threat effect is a reflection of a decisional, non-search process, then it should recur in a non-search version of the task.

## Experiment 3

In this final experiment a new sample of participants was tested in a partial replication of the classification task in Experiment 2. The images used in Experiment 2 were used here.

### Participants

Twenty-four naïve participants (mean age = 221, 20 female) were recruited from students of The University of York. There were three left-handed individuals. They received either a course credit or £4. All of the participants reported normal or corrected-to-normal vision.

### Results and Discussion

**FIGURE 5:**
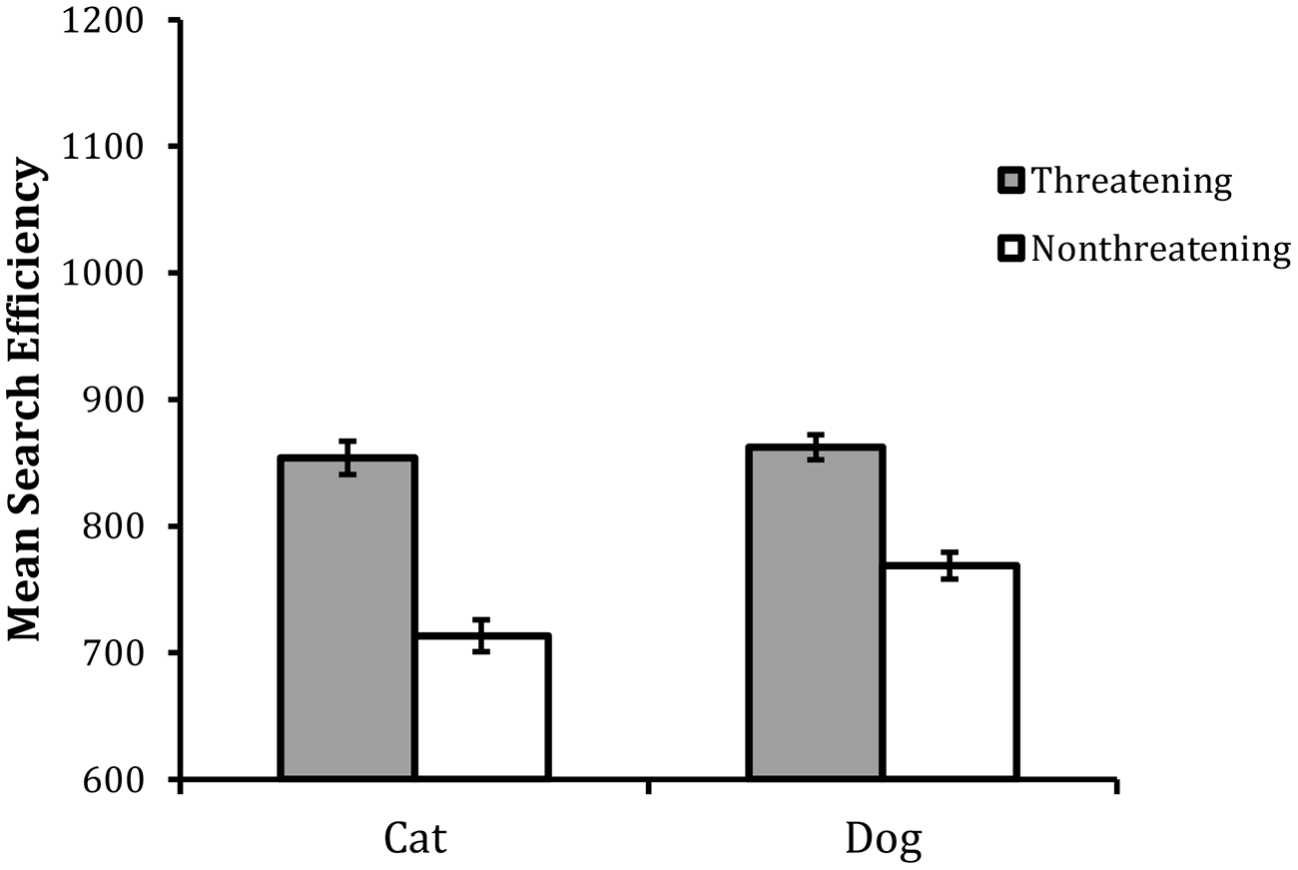
**Mean search efficiency scores as a function of target type in Experiment 3**. Error bars reflect within-participant SE after variation between participants was removed (after Bakeman and McArthur, 1996).

To maintain parity with the previous methods, the data were converted into IE scores. The summary data for the correct RTs and error rates are shown in **Table 5** and the summaries of the IE scores are shown graphically in **Figure 5**

**Table 5:** Mean RTs, SE, and mean percentage error rates (%E) for the various conditions of interest in the non-search classification task in Experiment 3.

Target type	Mean	SE	%E
Threatening cat	753	29	11.4
Threatening dog	775	30	9.9
Non-threatening cat	676	24	4.5
Non-threatening dog	733	23	4.3

The analysis revealed statistically significant main effects of valence, *F*(1,23) = 50.77, MSE = 6475, *p* < 0.001, and animal, *F*(1,23) = 5.74, MSE = 4241, *p*< 0.05. In addition the interaction between valence and animal was also statistically reliable, *F*(1,23) = 5.62, MSE = 2360, *p* < 0.05. An HSD test examining the nature of the interaction revealed that performance was overall worse with the threatening targets than the non-threatening targets and the size of this effect was larger in the cat target trials than the dog target trials. Although the difference between threatening cat and dog targets was not statistically reliable (*p*> 0.05), performance was overall best with the non-threatening cat targets (all *p*s < 0.05).

The central finding was that the classification of the threatening targets was less efficient than that of the non-threatening targets. This replicates the ‘reverse threat effect’ found in Experiment 2 and hence reveals a remarkably consistent pattern across the search and non-search versions of the classification task, a thorough examination of these findings is included in the “General Discussion.”

## General Discussion

The study began by examining performance in two variants of speeded ‘oddball’ visual search. In the detection tasks participants had to decide whether an image of a dog or a cat was present amongst non-target images of wild birds. On half of the trials no such ‘oddball’ image was present. In the classification task an oddball was always present and participants had to decide whether the target image was of a dog or a cat.

In Experiment 1, in the detection task, there was evidence of a threat advantage such that threatening images were detected more efficiently than non-threatening images. However, different patterns of performance occurred for the cat and dog images: the difference between performance with the threatening and non-threatening images of cats was greater than for threatening and non-threatening images of dogs. Clearly factors other than those associated with threat content were at play.

In the classification task the only effects of threat content arose with the images of cats but not dogs. Here again performance with the non-threatening cats is distinctive. The efficiency of classification decreased markedly as the display size increased. Looking across the data for the detection and classification tasks it seems that, for whatever reason, performance with the non-threatening cat images was relatively poor.

In attempting to understand the general principles at stake, it became evident that there were rather important stimulus differences across the threatening and non-threatening images: whereas the majority of the threatening images were headshots, the non-threatening images were less constrained. To address this possible confound, new images were sourced for the non-threatening instance so that the number of headshots were equated across the two stimulus kinds.

With this control in place the data from Experiment 2 revealed two important points. Firstly performance in both tasks was far more consistent than before, and, second, the effects relating to the difference between threatening and non-threatening images were expressed in terms of intercept rather than slope differences. The threat advantage in the detection task in Experiment 2 was considerably reduced to that reported in Experiment 1 and this is perhaps a testament to taking better control over incidental confounding stimulus factors. A consistent difference across the threatening and non-threatening images in Experiment 2 was presence of the salient snarl in the threatening images. According to the fear response hypothesis, this sort of threat cue should have been detected automatically (Öhman and Mineka, 2001). In contrast, there was no evidence in support of target pop-out, moreover the effects were more indicative of non-search response mechanisms than search processes. The data suggest that the threat effect in the detection data was more a reflection of responding to the threat cue than actually locating it in the display.

The classification data in Experiment 2 were also more consistent than in Experiment 1 and revealed an initially surprising reverse threat effect: the images of the non-threatening images were much easier to classify than the threatening images. Again this reverse threat effect was manifest in intercept rather than slope differences. The implication that this effect reflected a non-search process was tested in a final experiment. In Experiment 3 only a single image was presented and again the reverse threat effect obtained.

In sum when more careful stimulus controls were in place, relatively stable and consistent effects of image content have obtained. The picture that emerges is that the effects of ‘threat,’ such that they are, only become manifest once the target object has been located. There is no evidence that the search process was sensitive to the threat content of the oddball images. The suggestion is that the effects reflect the operation of response mechanisms and not the search processes. Participants in the detection task responded more efficiently to the threatening images than the non-threatening images. It has been suggested that this may well reflect some form of arousal elicited by the threatening stimuli, because independent ratings of the target images revealed that the threatening images were significantly more arousing than the non-threatening images. Such an idea accords well with the more recent writings of Lundqvist et al. (2014). In reviewing the speeded visual search literature on the processing of facial expressions, they concluded that a key determiner of performance was the arousal content of the target images and not the valence of target facial expression *per se*.

Although the reverse threat effect in the classification task in Experiment 2 was initially somewhat surprising, on reflection, this may well have a rather prosaic explanation. It is well known that more familiar and typical objects are, more easily classified than are less familiar and more atypical objects (Bültoff and Newell, 2006; Castelhano et al., 2008). In the present case it is highly plausible that the domestic cats and dogs were much more familiar to the participants than were the wild exemplars. Indeed it is also quite plausible that domestic cats and dogs are more typical, respectively, of cats and dogs than are the corresponding wild exemplars. On these grounds the reverse threat effect is nothing to do with threat at all but merely another demonstration of standard effects attributable to instance familiarity and/or typicality. Future work might address these issues by controlling for item familiarity whilst varying threat content. For instance, pictures of the same kinds of domestic cats and dogs could be sourced with examples of placid and snarling instances.

It is also not possible, on the basis of the data reported here, to draw firm conclusions about the predicted dissociation between detecting threat and identifying the cause of the threat. According to the fear response hypothesis, it should be possible to detect the presence of threat in advance of identifying its nature. It is clear that participants were generally more efficient in responding ‘present’ in the detection than they were in responding in the classification task. Some might take this as evidence for the fear response hypothesis. However, the data are more complex than this and have revealed that quite different factors are operating in the two kinds of tasks. Whereas it seems that the arousing nature of the images plays a key role in the detection task, it seems that item familiarity/typicality are key in the classification task.

There are many subtleties here that need to be considered before substantial progress can be made. For instance, it is relatively well established that speed of classification depends critically on the level of ‘classification’ judgment to be made. For example, performance in speeded object classification tasks has been shown to depend on the level of category being assigned. Classifying objects at the basic level (e.g., dog, cat, etc., Rosch, 1975) was initially shown to be easier than classifying objects at a more subordinate level (e.g., Poodle, Persian, etc.). Later work revealed that this could be reversed according to the expertise of the participant being asked to make the judgments (Tanaka and Taylor, 1991). In the present case concerns have been raised over the relative ease of assigning pictures of wild and domestic instances to the categories ‘DOG’ and ‘CAT.’ This leaves the question open as to whether some other form of classification would be more illuminating.

In this regard, future work is needed in order to address this issue directly and it may be that speeded visual search is not the ideal tool to use. Perhaps a more sensible option would be to adapt the paradigm described by Grill-Spector and Kanwisher (2005)? In their case, and on each trial, a single photographic image was presented briefly (image duration varied between 17 and 167 ms) and curtailed by a pattern mask. In different conditions participants were asked to make a detection, classification, or identification response to the image. In the detection task, half the time a scrambled object image was presented and participants simply had to indicate whether an image of an object had been presented. In the classification task, participants were instructed to respond with the basic level category name of the imaged object (car, house, flower etc.) and in the identification task they were instructed to respond with the subordinate category name (e.g., German Shepherd). Accuracy of report was mapped out as a function of image duration.

A surprising finding was that the functions for detection and classification were essentially the same, leading to the conclusion that “it takes no longer to determine an object’s category than to simply detect its presence” (Grill-Spector and Kanwisher, 2005, p. 159). Such a conclusion stands in stark contrast with the predictions of the fear response hypothesis. On these grounds it would be useful to use threatening and non-threatening images in the paradigm described by Grill-Spector and Kanwisher (2005) so as to test the fear response hypothesis directly.

## Conclusion

In closing, although the initial intentions were to uncover how threat content influences visual target detection and classification, some somewhat surprising evidence has emerged that implicates a number of different factors. As is clear from the contrasting patterns of effects across Experiments 1 and 2, the patterns of performance in these rather complex speeded search tasks are exquisitely sensitive to a range of stimulus factors that may or may not be under experimental control (cf. Quinlan, 2013). When more careful controls were adopted over image selection, the data revealed far more consistent and stable patterns of performance. For example, in both detection and classification tasks the effects of ‘threat’ were reflected in intercept rather than slope differences. On these grounds the effects appear to reflect more about non-search than search processes. Indeed it seems that the standard sorts of speeded visual search tasks that have been developed using very simple sorts of stimuli (such as colored shapes, Quinlan and Humphreys, 1987) may not be the ideal means by which to test the key ideas. Although the current results do not fundamentally undermine the fear response hypothesis, the evidence for the automatic detection of immediate visual threats remains illusive. Clearly separating effects of threat from those of arousal is also a major issue that needs to be more thoroughly examined (Lundqvist et al., 2014).

## Conflict of Interest Statement

The authors declare that the research was conducted in the absence of any commercial or financial relationships that could be construed as a potential conflict of interest.
